# Define and Conquer: How Semantics Foster Progress; A Response to Recent Commentaries

**DOI:** 10.15171/ijhpm.2017.116

**Published:** 2017-05-10

**Authors:** Wieteke van Dijk, Marjan J. Faber, Marit A.C. Tanke, Patrick P.T. Jeurissen, Gert P. Westert

**Affiliations:** ^1^Celsus Academy for Sustainable Healthcare, and Scientific Institute for Quality of Healthcare (IQ healthcare), Radboud Institute for Health Sciences, Radboud University Medical Center, Nijmegen, The Netherlands.; ^2^Scientific Institute for Quality of Healthcare (IQ healthcare), Radboud Institute for Health Sciences, Radboud University Medical Center, Nijmegen, The Netherlands.

The main message of our paper “Medicalisation and Overdiagnosis: what society does to Medicine” was: “*instead of solely a result of medicine, medicalisation and overdiagnosis consist of social cultural processes that take place both in and outside medicine.*”^1^ We were privileged to receive the thoughts and comments of three esteemed scholars on our perspective.^2-4^ Three authors approached the topic from a different angle and contributed to the discussions surrounding it. Hofmann stresses that overdiagnosis indeed is a likely candidate to be a social construct and calls for further research on this topic.^3^ Carter underlines the necessity of conceptually rigorous, well-reasoned and empirically sound work on this important subject. She also presents a meaningful definition of overdiagnosis, which she developed with colleagues.^2^ Wardrope explains how a definition or model is always a proxy for reality and should not be expected to serve purposes out of its original territory.^4^ Although very different in approach and emphasis, in fact all three address important questions: how to better understand and define medicalisation and overdiagnosis, and how to proceed into meaningful future research?

We agree with most of the arguments brought forward and thank the authors for explicating them. However, one aspect of Wardrope’s contribution puzzled us. Wardrope’s central message is that apart from ‘what society does *to* medicine,’ we should also pay attention to ‘what society does *with* medicine.’^4^ According to our best understanding of his argument, the key underlying argument is that the connection we made between society and medicine cannot be contributed to medicalisation. Wardrope adds some relevant insights to the discussion and we welcome a discussion on ‘what society does *with* medicine.’ Nonetheless, we disagree that this phenomenon cannot be aligned with medicalisation. Wardrope does not state explicitly how he defines medicalisation. However, his implicit definition of medicalisation excludes the possibility of a relation between society and medicine. To further explore this, we will first briefly summarize Wardrope’s arguments, after which we will present our comments.

Wardrope explains how biomedical models, like any *fictional construct*, are designed to meet specific, biomedical purposes, and cannot be extended far from this purpose without consideration. Biomedical models and diagnoses reify complex biological systems for a purpose: to aid the understanding and treatment of *medical* problems. He uses the metaphor of a map: a useful yet simplified version of a geographic area. A problem arises when the map is mistaken for the territory that it represents. This is the case when the use of biomedical labels is stretched outside the realm of medicine, when society applies medical labels in a far broader manner than in which the original construct was meant to be applied. Society uses medical labels and diagnoses to in- or exclude people or to grant welfare benefits. Therefore, it is not the medical profession which is medicalising social problems, it is society stretching the boundaries of diagnoses to fields unrelated to medicine. Medical professionals do realise this limitation of the medical model, and are conscious of the cultural and situational aspects of medical labels. Apparently, legislators and other societal actors do not recognise these boundaries as clearly. This is the summary of what society does *with* medicine. In order to lessen this problem, the limitations of the biomedical image of the world should be openly acknowledged, and society and medical professionals should be more open to other perspectives to view the world.

According to Wardrope, medicalisation invariably entails that medical professionals individualise problems. Medical professionals are conscious of the limitations of medical labels, therefore medicalisation cannot be aligned with societal influence on medicine. This linkage is well expressed in the following quote from Wardrope’s commentary: **“***This cycle is driven by the move from medicalisation to the interpretation of human experience overwhelmingly in medical terms; but if the above argument is correct, the latter is not an inevitable consequence of the former***”** (p.3).

We do not regard this ‘move’ incompatible with medicalisation. What defines medicalisation, other than “*the interpretation of human experience overwhelmingly in medical terms*”? We show in a recently performed scoping review (submitted for publication) that the definition of medicalisation varies across studies. Various definitions co-exist, and each definition highlights different aspects of medicalisation. The involvement of the medical profession, which Wardrope seems to regard as necessary for medicalisation to occur, is not necessarily part of every definition. Recent scholars underline the possible involvement of non-medical groups, such as consumers or commercial parties.^5^ What is essential is the explanation of human problems in medical terms. Nonetheless, we want to stress that whether one regards this stretching of biomedical labels by society as medicalisation, depends on how one defines medicalisation. This may seem axiomatic, but we agree with Carter that *“definitional work is not for its own sake, it has consequences.”*^2^ While every definition is a proxy for reality, the territory one can cover in research depends on how both map and territory are defined.

In conclusion and in accordance with the three commenting authors, we want to emphasize that future research on medicalisation and overdiagnosis should start from a clear and well-developed definition of the subject under study. This extends beyond semantics because it helps us interpret and understand the society we study as well as the work of others.

## Ethical issues

Not applicable.

## Competing interests

Authors declare that they have no competing interests.

## Authors’ contributions

WvD conceptualised the article, all others provided input and suggestions on subsequent version.

## Authors’ affiliations

^1^Celsus Academy for Sustainable Healthcare, and Scientific Institute for Quality of Healthcare (IQ healthcare), Radboud Institute for Health Sciences, Radboud University Medical Center, Nijmegen, The Netherlands. ^2^Scientific Institute for Quality of Healthcare (IQ healthcare), Radboud Institute for Health Sciences, Radboud University Medical Center, Nijmegen, The Netherlands.
